# An educational campaign to increase chiropractic intern advising roles on patient smoking cessation

**DOI:** 10.1186/1746-1340-14-24

**Published:** 2006-10-12

**Authors:** Marion W Evans, Cheryl Hawk, Sheryl M Strasser

**Affiliations:** 1 Parker College of Chiropractic Research Institute, 2500 Walnut Hill Lane, Dallas, Texas 75229, USA; 2 Vice-President of Research and Scholarship, Cleveland Chiropractic College, 6401 Rockhill Road, Kansas City, Missouri 64131, USA; 3 Adjunct Faculty, The University of Alabama Health Sciences Department, 214 East Annex, Box 870311, Tuscaloosa, Alabama 35487-0311, USA

## Abstract

**Background:**

Tobacco use, particularly smoking, is the most preventable cause of death in the United States. More than 400,000 premature deaths are associated with its use and the health care costs are in the billions. All health care provider groups should be concerned with patients who continue to smoke and use tobacco. The US Preventive Services Taskforce and Health People 2010 guidelines encourage providers to counsel smokers on cessation. Current studies, though limited regarding chiropractic advising practices indicate a low engagement rate when it comes to providing cessation information.

**Objective:**

To test a campaign regarding initial impact aimed at increasing chiropractic interns advising on cessation and delivery of information to smokers on cessation.

**Discussion:**

Chiropractic interns do engage patients on smoking status and can be encouraged to provide more cessation messages and information to patients. The initial impact assessment of this campaign increased the provision of information to patients by about 25%. The prevalence of smoking among chiropractic patients, particularly at teaching clinics may be lower than the national averages.

**Conclusion:**

Chiropractic interns can and should be encouraged to advise smokers about cessation. A systematic method of intake information on smoking status is needed and a standardized education protocol for chiropractic colleges is needed. Chiropractic colleges should assess the adequacy of their advising roles and implement changes to increase cessation messages to their patients as soon as possible.

## Background

Tobacco use, particularly smoking, is the most preventable cause of death in America. It causes more than 400,000 premature deaths a year, and the direct health care costs exceed $150 billion annually [1]. It is well established that all health care provider groups can, and should, advise smoking patients on cessation [2]. The United States Preventive Services Task Force, Healthy People 2010 and The Centers for Disease Control and Prevention suggest that all clinical providers counsel smoking patients on cessation and offer an opportunity to quit [2-4]. Ahluwalia and colleagues suggest that tobacco use status be considered a 5^th ^vital sign, due to its impact on health [5].

Studies indicate that US medical and chiropractic school curricula are deficient in training their interns to assume advising roles in the area of smoking cessation [6-9]. Currently, approximately 20% of the U.S. population smokes [10]. However, a minority of tobacco users report having been advised to quit, and even fewer have been given specific information on how to quit [2]. Doctors of chiropractic see about 10% of the U.S. population annually but see approximately 30% of back pain patients [11,12]. Among those with chronic spine conditions, smoking is often the number one co-morbidity reported [13]. Rechtine and colleagues conducted a successful smoking cessation campaign in an orthopedic spine center where some 90% of post-surgical failures and infections were found to be in smokers [14]. Spine surgeons do not typically serve in a primary care capacity; however, Rechtine and colleagues successfully increased cessation rates from 19% of patients to 35% in their study. It is clear that since smoking and tobacco use affects every body system, all providers, not only those in primary care, need to be concerned with the patients' tobacco use.

Hawk and others report that chiropractors self-report a high level of involvement in health promotion activities in their practices [15]. Hill suggests practitioners who practice complementary and alternative medicine (CAM) may be most appropriate for delivery of health promotion messages as they are already seen as holistic by patients and lean conceptually toward prevention [16]. The involvement of chiropractors in patient smoking cessation counseling has not been thoroughly explored. However, Hawk and Evans, through an investigation of 9 chiropractic teaching clinics in the US, found the minority of smoking patients (39.7% or 52/131) had been advised on quitting and even fewer (18% or 24/131) had been given specific information on quitting [9].

This article reports the initial impact of an educational campaign aimed at increasing chiropractic interns' provision of smoking cessation advice to teaching clinic patients. The purpose of the intervention was to increase interns' smoking cessation advising as measured by a pre- and post-intervention patient surveys on whether they had been asked about smoking status, and if a smoker, if they received advice and materials from their intern.

## Methods

This was an educational intervention aimed at chiropractic interns following a pre-test/post-test design using independent patient samples. The education intervention was delivered to chiropractic interns at a chiropractic college teaching clinic in Dallas, Texas in the Fall of 2005. Outcomes were evaluated by comparing the proportion of smokers in pre- and post-intervention patient samples who reported that their interns had asked about their smoking status on their last visit, including advice on cessation, including written cessation information.

A second survey examined interns' participation levels and motivation factors associated with smoking cessation counseling by interns.

### The Education Campaign

The education campaign aimed at interns involved 7 main components. These included both instructional materials to reinforce intern training and informational materials to reinforce the smoking cessation message to clinic patients. Role play opportunities were also utilized and exchanges of ideas on how to advise patients that had been performed by interns were explored as well. The development of these materials is described elsewhere [17]. The tailored educational materials included several components:

#### Instructional Materials for Interns

1) 1 hour Power Point lecture given by the principal investigator.

2) 3" × 5" card guiding interns through engagement of patients using the Surgeon General's 5-A's [2]

3) Stamp for clinic supervising doctors to track smoking cessation advising done by interns.

#### Informational Materials for Patients

1) Campaign buttons for the intern's clinic jacket.

2) Posters placed in each treatment of report room of the outpatient clinic from the CDC.

3) Brochure rack at the clinic check-out desk to provide quick and easy access to brochures for patients.

4) Resource directory of cessation programs available in the Dallas/Ft. Worth Metroplex.

### Patient Sample

Participation in the patient survey was offered to every established patient who entered the Dallas outpatient teaching clinic for 5 consecutive days. Inclusion criteria were:

1) Having been seen in the clinic in the prior 10 working days and

2) 18 years of age or older.

3) Not having participated in the study performed by Hawk and Evans [9] (determined by report) one year prior.

Patients who were queried in the second round of surveys were independent of the pre-intervention group of patients.

### Intern Sample

Following patient data collection, interns were queried as to their participation levels. Inclusion criteria for interns were:

1) In the 7^th ^or 8^th ^trimester of study, as these groups attend classes on campus and also treat patients.

2) Active engagement in treating patients in the outpatient clinic that semester.

Trimester 9 students were excluded, since many had already met clinic requirements and none had active classroom participation requirements on campus on a regular basis.

### Survey Instrument Development and Administration

**Figure 1:**
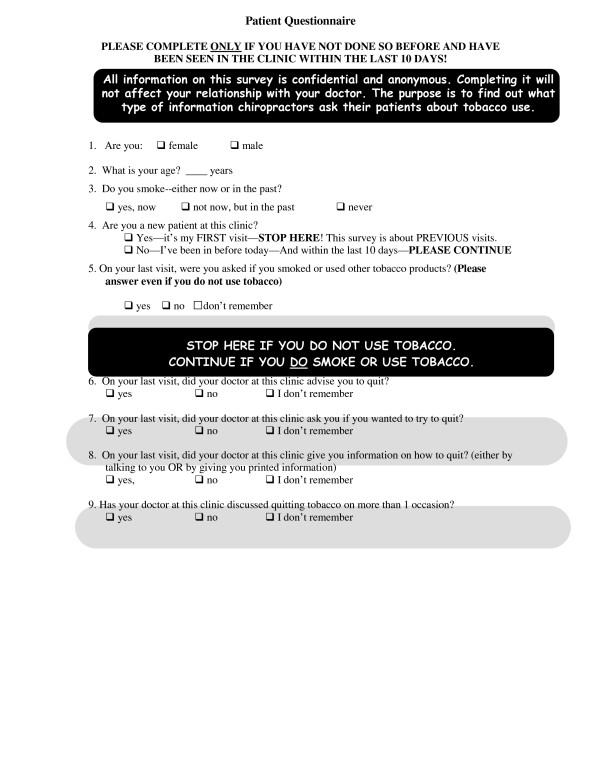
Survey instrument used to survey patients in pre and post-intervention groups.

A modified patient survey developed by Hawk in 2004 was utilized in this study [4]. Changes to the original survey included a focus on smoking behavior rather than general tobacco use and was based on scientific literature regarding smoking-cessation counseling. The instrument had been tested for face validity and user-friendliness prior to the study by Hawk and Evans and again after slight modifications were made prior to this study. The survey consisted of one page of 9 questions and was designed to take less than three minutes to complete [Fig 1]. The survey was administered to patient in the waiting area following registration.

**Figure 2:**
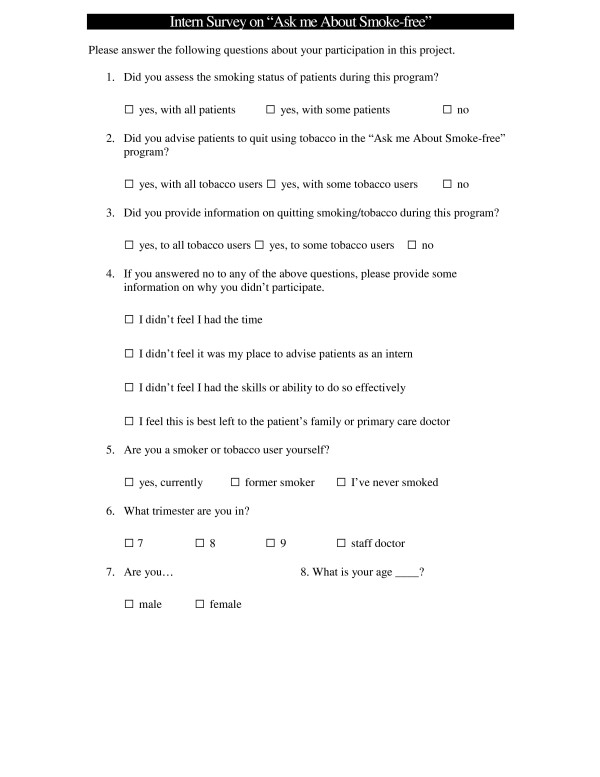
Survey used in surveying interns participation levels.

The intern survey was developed for post-education assessment of intern participation levels. This survey was a one page survey containing 7 questions [Fig 2]. It was designed to take less than three minutes to complete. The survey was tested for face validity among scientists as the Parker Research Institute and three other health scientists in the health promotion field. Changes were made to make the instrument user friendly with students.

### Data Analysis

Comparisons of data were made initially with chi square analysis for categorical variables and t-testing for the only continuous variable, age [Table 3]. Logistic regression was used to evaluate possible predictors for continuous and categorical variables with categorical outcomes. The following continuous and categorical variables from the patient survey were tested: gender, age, use of tobacco, asked about smoking status on last visit, advised to quit smoking on last visit, given information on cessation on last visit, and was quitting discussed on more than one visit with the patient if they were a smoker.

**Table 3:** Summary of Crosstabulations of Variables Regarding Gender, Trimester, Smoking Status, Advising and Giving Information Categories and Intern Groupings

Variable	No (%)	Yes (%)	χ^2^
Smoke3			
-Male	37.5	62.5	.186
-Female	22.0	78.0	
			
-Tri7	62.0	38.0	.06
-Tri8	85.0	15.0	
			
Smoker			
-Male	90.0	10.0	.09
-Female	27.0	0.0	
			
-Tri7	93.6	6.4	.827
-Tri8	95.0	5.0	
			
Assessed2			
-Tri7	60.0	40.0	.228
-Tri8	75.0	25.0	
			
-Smoker			
-Smoker	63.5	36.5	.642
-Non/Fmr	75.0	25.0	
-Smoke3			
-Smok/Fmr	58.7	41.3	.166
-Non	76.2	23.8	
			
Gender2			
-Male	70.0	30.0	.226
-Female	56.0	45.0	
			
Advised2			
-Tri7	77.8	22.2	.559
-Ti8	84.2	15.8	
-Smoker			
-Smoker	50.0	50.0	.127
-Non/Fmr	81.7	18.3	
-Smoke3			
-Smok/Fmr	80.0	20.0	.967
-Non	79.5	20.5	
			
Gender2			
-Male	82.0	18.0	.557
-Female	75.0	25.0	
			
Gavequit			
-Tri7	85.0	15.0	.982
-Tri8	85.0	15.0	
			
-Smoker			
-Smoker	50.0	50.0	.045*
-Non-Fmr	87.1	12.9	
-Smoke3			
-Smok/Fmr	80.0	20.0	.469
-Non	87.0	13.0	
			
Gender2			
-Male	90.0	10.0	.456
-Female	80.0	20.0	

*p < .05 Note: Smoker variable = smoker v.s. non/former smoker, Smoker3 = smoker/former v.s. non-smoker, "2" following variable indicates recode to dichotomize negative and "I don't remember" categories.

For analysis of the intern data, the following continuous and categorical variables were tested: gender, age, was smoking status [of your patient] assessed during the campaign, did you advise smokers to make a quit attempt, did you provide information to smoking patients, and reasons why you did not participate. Trichotimized variables were collapsed into 2 categories.

## Results

### Demographics

#### Patient Demographics

There were a total of 538 usable surveys completed by patients in the Dallas clinic. A majority of patients completed a survey as all patients meeting the inclusion criteria were offered a survey and only 5 patients who met the criteria refused to complete one. Reasons for refusal included being in too much pain or aversion to surveys. The pre-intervention survey was completed by 342 individuals and 212 completed the post-intervention survey. The pre-intervention group 6 surveys were unusable either due to patient age or because a non-smoker completed the additional smoker-oriented questions on the survey by mistake. In the post-intervention group 6 surveys were considered unusable for the same reasons and an additional 4 were excluded from the study analysis because respondents identified themselves as past-smokers; however, they also completed the survey section designated for current smokers. Of the usable surveys, 475 (88.3%) patients did not smoke. The mean age of patients in the sample was 44.4 years with a rage of 18 to 84 years. Males, (n = 283) had a mean age of 45 (SD = 13.82) and females, (n = 255) had a mean age of 43.8 (SD = 15.19). Data were considered fairly normal in distribution but had bimodal peaks noted at the 25–30 age range and 50–55. There was no statistical difference in the mean age of smokers (43 years) and non-smokers (45 years). There were a total of 63 smokers (11.7%) with 41 being male (65% of smokers) and 22 females (35%). Of the 41 male smokers, 24 were in the pre-intervention group and 17 were in the post group. While females made up only 35% of smokers in the study population, there were only 13 in the pre-intervention group (21% of smokers in the study) and 9 in the post-intervention group (14.3% of smokers in the study). Additional demographic characteristics on patients in the survey are included in Table 1.

**Table 1:** Demographic Characteristics of Patient Participants

	Pre		Post	
Characteristic	no.	%	no.	%

Gender				
Male	173	51.5	110	54.5
Female	163	48.5	92	45.5
				
Smoking Status				
Smoker	37	11.0	26	12.9
Non-Smoker	299	89.0	176	87.1
				
Smoking Status				
Within Gender				
Male				
Smoker	24	13.9	17	15.5
Non-Smoker	149	86.1	93	84.5
Female				
Smoker	13	8.0	9	9.8
Non-Smoker	150	92.0	83	90.2

#### Intern Demographics

There were 179 interns and 68 total surveys returned. One survey was unusable as the intern was not seeing patients that semester, for a total of 67 usable surveys (38% participation rate). The mean age of students in the intern survey was 29.3 years (range 22–48 years). Forty (60%) of the interns were males and 27 (40%) were females. Of the interns in the study, only 4 (6%) were current smokers and 17 (25%) were former smokers, 46 (69%) having never smoked. The current smokers (n = 4) were all males.

#### Patient Data

Regarding patient surveys and whether patients would report differences in smoking status and subsequent intern engagement based on demographics, the following results were found after analysis on χ^2 ^testing; there was a significant difference in the number of male versus female smokers in the study in aggregate. There were 242 males who did not smoke (85.5%) and 233 females (91.4%) Forty-one males reported smoking (14.5%) and 22 females (8.6%). This difference was significant at *p *< .035 indicating almost twice as many males smoking as females. Gender breakdown in pre versus post grouping was not significantly different. There were 173 males in the pre group and 163 females and there were 110 males in the post-group with 92 females (*p *= .504).

Regarding patients' reports of being asked about smoking status, receiving advice to quit if they smoked, and being asked if they wanted to try to quit, no significant difference in rates were noted among those in the pre-intervention groups when compared to post-intervention participants based on patient demographics. Table 2 lists χ^2 ^statistics for these analyses and includes the χ^2 ^for smoking and gender as well.

**Table 2:** Summary of Crosstabulations of Variables Regarding Gender, Smoking Status, Advising and Information Giving Categories and Pre/Post Groupings

Variable	#No (%)	#Yes (%)	χ^2^
SmokeY/N			
Male	242 (85.5)	41 (14.5)	.035*
Female	233 (91.4)	22 (8.6)	
			
Gender			
Pre-Group Male	163 (48.5)	173 (51.5)	.504
Post-Group Male	92 (45.5)	110 (54.5)	
			
Asked Patients About Smoking Status			
Pre-Group	267 (79.5)	69 (20.5)	.126
Post-Group	149 (73.8)	53 (26.2)	
			
Advised Patients on Cessation			
Pre-Group	19 (51.4)	18 (48.6)	.423
Post-Group	16 (61.5)	10 (38.5)	
			
Asked if Patient Wanted to Try Quitting			
Pre-Group	26 (70.3)	11 (29.7)	.469
Post-Group	16 (61.5)	10 (38.5)	
			
Gave Patient Cessation Info			
Pre-Group	35 (94.6)	2 (5.4)	.007*
Post-Group	18 (69.2)	8 (30.8)	
			
Discussed Quitting on More Than One Occasion			
Pre-Group	19 (51.4)	18 (48.6)	.916
Post-Group	13 (50.0)	13 (50.0)	

*p < .05

Regarding smokers who reported being given information on cessation there was a significant difference in rates among smokers in the pre-intervention and post-intervention groups on χ^2 ^testing. Among pre-intervention smokers (n = 37) only 2 (5.4%) said they had been given specific information on cessation. Among those smoking patients in the post-intervention group (n = 26), 8 (30.7%) said they were given information on cessation. This was significant at the .007 level with an odds-ratio of 7.8. Although numbers are small, this represents an increase in over 25% within one month and those in the post-intervention group were about 8 times more likely to have been given information on cessation.

When smokers were asked if they had been advised to quit on more than one occasion, about 50% in both pre and post-intervention groups answered in the affirmative.

### Predictors of Information Being Given to Patients

In logistic regression modeling only the pre/post grouping was significant when age, gender and pre/post group tested. Patients in the post-intervention group were about 8 times more likely to report being given information than the pre-intervention patient group (*p *= .014, OR = 8.196, 95% CI 1.54, 43.5).

#### Interns' Reported Reasons for Not Participating in the Campaign

When interns did select a reason for not participating in the campaign on the intern survey, (n = 16), 9 said they didn't have time, 4 said it was not their place to advise smoking patients and only 3 said they didn't feel they had the skills.

## Discussion

The purpose of this study was to determine if an education campaign, delivered to chiropractic interns, could have an initial impact on intern's advising roles with patients regarding assessment of smoking status and smoking cessation information. We believe the results generally support the idea that interns can be trained to assess smoking behaviors and deliver information to those patients on the importance of cessation. Intern demographics may, or may not, affect the rates of the advising roles. There are certain limitations of this study. First, it is an impact study to measure short-term changes in intern behavior, not an outcome study. Another limitation was that both patients and interns were essentially a convenience sample. Although patients were technically a consecutive sample, neither sample may be representative of the populations they are suggested to represent.

Among patient participants all were patients at an outpatient teaching clinic at Parker College of Chiropractic in Dallas, Texas. Even though they ranged in age from 18 to 84 (mean = 44) they differed in smoking status from current US prevalence statistics. According to the CDC, as of 2004, 20.9% of the US citizens were current smokers. [10] In our sample, only 11.7% were current smokers. The smoking prevalence for males and females in the sample did not have the same characteristics as the US population either. Current MMWR data cited here [10] stated that 23.5% of US males smoked and 18.5% of females. However, in the study sample, 14.5% of males smoked and only 8.6% of females. They may be representative of other patients seen in chiropractic teaching clinics as Hawk and Evans [9] reported 16% of participants in a study of 9 teaching clinics in the US were current smokers. Essentially, patients at the Parker College of Chiropractic clinic in Dallas smoked at a rate 40% lower than the current US prevalence rate.

Interns in the sample (n = 67) seem typical regarding age and gender categories but when it comes to smoking prevalence, they do not smoke at a rate near the current US prevalence. Among all interns completing the survey, only 4 (6%) were current smokers and there were no current female smokers in the sample. The number of eligible interns who declined participation may have influenced this prevalence rate and there could have been reporting bias reflected in the sample as well. A majority of interns did not complete a survey.

Regarding patient responses on whether they were asked about smoking status on their last visit, many wrote on their survey that they had never smoked and although their intern did not ask on the last visit, they did ask about smoking status on the patient's first visit and therefore, the patient felt there was no reason that the intern would have asked this again. Interns, by the same token, often wrote on their survey that they would have participated in the campaign but had no smoking patients in their patient-base. With only 11.7% of patients in the sample reporting smoking, this seems reasonable and therefore, rates of participation were likely affected by this fact.

Regarding patients who reported being advised to quit on more than one occasion, about 50% said they had been told to in both the pre and post-intervention groups. This is slightly better than what has been reported on engagement by primary care physicians [6,7]. However, intern engagement levels could improve with time should a cessation education campaign become institutionalized at the college. Only a few interns felt it was not their job to counsel patients on smoking cessation and only a few said they didn't have the skills or time.

## Conclusion

Generally, numbers were small in this sample when it came to those who smoked – both interns and patients. A larger sample of patients and interns, perhaps across the country and repeating this campaign at several campuses could yield more pertinent information on this topic. We suggest this be considered.

The analysis of this data indicates that in a short period of time some impact can be made on intern behaviors regarding cuing patients to stop smoking. If cessation information is made easily available to interns, we believe they will provide it to their patients. Although not all results were encouraging and the number of smokers in the study population was small, information was given to smoking patients at a higher rate after the education intervention. This is the area where the ball is typically dropped. Most smoking patients have not been given specific information on cessation by their doctor [2,6,7,18]. Therefore, we assume that a broader, integrated campaign would have enhanced effects on interns' advising behaviors. However, this needs to be investigated further. The development of a standardized delivery mechanism for smoking cessation education for interns is much needed. Clearly, there is a need for this to be a standard part of chiropractic education. Moving interns to adopt this practice must begin in the curriculum and clinical competencies of the colleges.

This campaign was inexpensive and was well-received at Parker College of Chiropractic. It has now been integrated into the curriculum as part of the wellness class required in trimester 5 prior to interns seeing patients in student or outpatient clinics. It has been popular and generated a request for a smoke-free campus which will take effect in January 2007 with full faculty and board support.

During the month-long campaign, 4 patients and 1 intern self-reported quit attempts to the principal investigator. If every chiropractic college and teaching clinic would integrate smoking cessation advising into the curriculum and clinic competencies of their colleges, we have no doubt that a significant effect would be seen in the amount of information provided to patients.

Chiropractic has been long seen as a holistic profession that says it emphasizes wellness and better health for patients. We see no reason advising smoking patients on cessation should not be a part of routine clinical practice of chiropractic and feel it should be made a requirement in all chiropractic colleges.

## List of Abbreviations

CAM-complementary and alternative provider

CDC-United States Centers for Disease Control and Prevention

5-A's-United States Surgeon General's 5-A's for advising smokers of cessation

n-number

p-p-value

SD-standard deviation

US-United States

χ^2^-chi square

## Competing interests

The author(s) declare that they have no competing interests.

## Authors' contributions

ME developed the general study design, collected, manipulated and analyzed the data, and prepared the manuscript. CH assisted in the study design, developed the patient survey and assisted in development and revision of the manuscript. SS assisted in the study design and in the development and revision of the manuscript. All authors read and approved the final manuscript.
